# Maternal betaine supplementation affects fetal growth and lipid metabolism of high-fat fed mice in a temporal-specific manner

**DOI:** 10.1038/s41387-018-0035-z

**Published:** 2018-05-24

**Authors:** Yaelle Joselit, Khatia Nanobashvili, Chauntelle Jack-Roberts, Esther Greenwald, Olga V Malysheva, Marie A Caudill, Anjana Saxena, Xinyin Jiang

**Affiliations:** 1 Departments of Health and Nutrition Sciences, Brooklyn College of City University of New York, Brooklyn, NY 11210 USA; 2 Division of Nutritional Sciences, Cornell University, Ithaca, NY 14853 USA; 3 Departments of Biology, Brooklyn College of City University of New York, Brooklyn, NY 11210 USA

## Abstract

**Background/objectives:**

Maternal obesity increases the risk of gestational diabetes mellitus (GDM), which results in fetal overgrowth and long-lasting metabolic dysfunctioning in the offspring. Previous studies show that maternal choline supplementation normalizes fetal growth and adiposity of progeny from obese mice. This study examines whether supplementation of betaine, a choline derivative, has positive effects on fetal metabolic outcomes in mouse progeny exposed to maternal obesity and GDM.

**Methods:**

C57BL/6J mice were fed either a high-fat (HF) diet or a control (normal-fat, NF) diet and received either 1% betaine (BS) or control untreated (BC) drinking water 4–6 weeks before timed-mating and throughout gestation. Maternal, placental, and fetal samples were collected for metabolite and gene-expression assays.

**Results:**

At E12.5, BS prevented fetal and placental overgrowth and downregulated glucose and fatty acid transporters (*Glut1* and *Fatp1*) and the growth-promoting insulin-like growth factor 2 (*Igf2*) and its receptor *Igf1r* in the placenta of HF, glucose-intolerant dams (*P* < 0.05). However, these effects disappeared at E17.5. At E17.5, BS reduced fetal adiposity and prevented liver triglyceride overaccumulation in HF versus NF fetuses (*P* < 0.05). BS fetal livers had enhanced mRNA expression of microsomal triglyceride transfer protein (*Mttp*) (*P* < 0.01), which promotes VLDL synthesis and secretion. Although we previously reported that maternal choline supplementation downregulated mRNA expression of genes involved in de novo lipogenesis in fetal livers, such alterations were not observed with BS, suggesting differential effects of betaine and choline on fetal gene expression.

**Conclusion:**

We propose a temporal-specific mechanism by which maternal BS influences fetal growth and lipid metabolic outcomes of HF mice during prenatal development.

## Introduction

Betaine (*N*,*N*,*N*-trimethylmethanaminium) is a methyl derivative of glycine which is formed from glycine and three methyl groups. Betaine is naturally found in foods such as shrimp, beets, and whole grains^1^. It can also be derived from the semi-essential nutrient choline (*N*,*N*,*N*-trimethylethanolammonium) in the body via oxidation mediated by choline dehydrogenase (CHDH). Betaine serves as an osmolyte, a methyl donor, and a lipotrope, and is primarily found in the livers of mammals.

The interaction of betaine with energy and macronutrient metabolism has been revealed in multiple studies. Betaine is used as an additive to animal feed to generate a leaner carcass^2^. Betaine treatment in cells enhances mitochondrial and cellular respiration, mitochondrial potential, and ATP production, thereby increasing energy expenditure^3^. Like its precursor choline, betaine supplementation (BS) prevents fatty liver and hepatic damage in rodents^4–8^, possibly in part by upregulating the systemic metabolic regulator fibroblast growth factor (Fgf) 21 or altering the DNA methylation of genes involved in lipid metabolism. Recent studies also suggest that BS in mice improves their glucose tolerance and reduces insulin resistance^5, 9^. Betaine is also available as a nutrition supplement, although its clinical effect on non-alcoholic fatty liver disease is not clear^10^.

The importance of betaine during prenatal and early postnatal development has attracted immense research interest. Betaine has the ability to donate a methyl group to convert homocysteine to methionine. Methionine can then be converted to *S*-adenosylmethionine (SAM) which donates a methyl group for DNA and protein methylation. Prenatal BS in sow modifies DNA methylation and expression of genes related to cholesterol metabolism and gluconeogenesis in the liver of piglets^11–13^.

However, the influence of betaine on maternal and fetal endpoints remains to be determined when maternal energy supply is in excess. It is consistently shown that high-fat (HF) fed mice become obese before gestation and develop glucose intolerance during gestation, resembling maternal obesity and gestational diabetes mellitus (GDM) in humans^14, 15^. The increased nutrient transport through the placenta leads to fetal overgrowth and excess adiposity^16–18^. Our previous studies suggest that prenatal supplementation of choline in HF mice prevents fetal overgrowth at E12.5 and excess adiposity at E17.5, possibly via the reduction in placental glucose and fat transport or fetal hepatic lipogenesis^15, 19^. As the oxidized derivative of choline, maternal BS may have similar effects to choline supplementation (CS) on methyl group donation and epigenetic regulation. However, betaine cannot be converted back to choline (i.e., oxidation of choline to betaine is an irreversible process), and thus may have a differential influence on lipoprotein structure as well as lipid transport than choline which is an essential component on the phospholipid membrane of lipoproteins.

The growing epidemic of maternal obesity increases the incidence of GDM to as high as 15% in some populations^20–22^, which also increases the risk of fetal overgrowth or macrosomia (i.e., over 4 kg at birth)^16–18^. Calorie control and weight management during pregnancy do not consistently normalize fetal weight^23–27^. Betaine has the potential to influence nutrient transport and epigenetic regulation of fetal genes, which may subsequently normalize fetal growth and metabolism. The current study examines the effect of maternal BS on outcomes of maternal obesity and GDM in mouse progeny. In this study, we can assess whether choline and betaine act within the same metabolic pathway, through DNA methylation, or whether they pose unique outcomes depending on their different roles as a membrane structural component, an osmolyte, or a mitochondrial respiration modifier.

## Methods

### Animals and diets

**Fig. 1:**
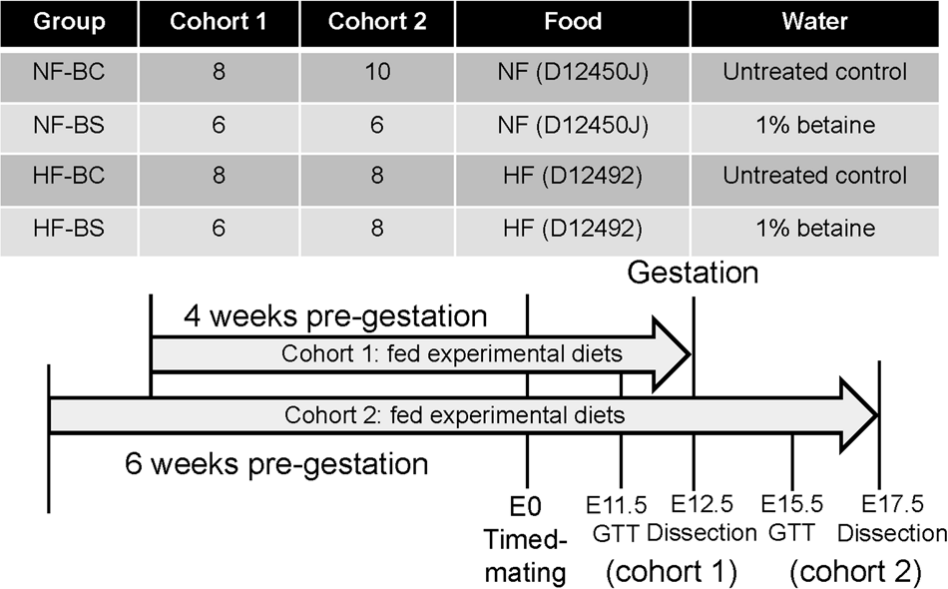
Design of the study. Female C57BL/6J mice were divided into four groups and fed with the normal fat (NF) no betaine (BC) diet, NF betaine-supplemented (BS) diet, HF-BC diet, or HF-BS diet for 4 (cohort 1) or 6 (cohort 2) weeks before timed-mating and throughout gestation. Male mice follow the NF-BC diet until timed-mating. The intraperitoneal glucose tolerance test (GTT) was conducted on embryonic day (E) 11.5 and 15.5. Dissection was conducted on E12.5 (cohort 1) or E17.5 (cohort 2). *n* is the number of dams in each group from which data and samples were collected

Six-week-old C57BL/6J mice were obtained from Jackson Laboratory. The mice were housed at 22 °C, humidity 40–60%, and 12-h light/dark cycle with regular bedding and enrichment. The female mice were randomly divided into four groups: the normal diet control (NF-BC) group received a normal diet (D12450J, Research Diets, New Brunswick, NJ, USA) containing 10% kcal from fat and untreated drinking water; the NF betaine-supplemented (NF-BS) group received the normal diet and purified drinking water supplemented with 1% betaine anhydrous; the high-fat control (HF-BC) group received a HF diet (D12492, Research Diets) containing 60% kcal from fat and untreated drinking water; and the HF betaine-supplemented (HF-BS) group received the HF diet and purified drinking water supplemented with 1% betaine anhydrous (Fig. 1). Male mice for mating received the NF-BC diet and purified drinking water. All animals had free access to food and water. Composition of the two diets has been described previously^15^.

In cohort 1, mice were fed 4 weeks of the experimental diets after which two female mice and one male mouse were caged together for timed-mating. The presence of a vaginal plug indicated mating and was recorded as embryonic day (E) 0.5. Female mice continued to receive their assigned diets during gestation until dissection at E12.5.

In cohort 2, pre-pregnancy feeding of the experimental diets was extended to 6 weeks to attain greater maternal obesity. The female mice underwent timed-mating and received experimental diets during gestation until dissection at E17.5, a late gestational time point (Fig. 1). There were at least six dams in each group. Sample size selection was based on previous studies showing differences in fetal growth due to HF feeding. For both cohorts, animals were weighed every week to assess weight gain throughout the study. Food and water consumption was measured each week. The NF and HF diets do not contain betaine. Supplemental betaine was calculated as the concentration of betaine in water multiplied by the volume of water consumed each week.

### Intraperitoneal glucose tolerance tests

Intraperitoneal glucose tolerance (IGT) tests were conducted on the female mice at E11.5 for cohort 1 and at E15.5 for cohort 2 as previously described^15^. Blood glucose levels were checked at baseline (0 min), 15, 30, 60, 90, and 120 min after glucose injection. The area under the curve (AUC) was calculated^28^.

### Sample collection

Animals were killed at E12.5 and E17.5 for cohorts 1 and 2, respectively by carbon dioxide inhalation. Immediately after euthanasia, maternal blood was collected into a serum separator tube (BD, Franklin Lakes, NJ, USA) following cardiac puncture to obtain serum. Maternal liver, abdominal fat, placentas, and fetuses were collected, rinsed in phosphate-buffered saline, and dried on absorbent paper. These samples were first weighed on an analytical balance. Subsequently, they were either flash frozen in liquid nitrogen and stored at −80 °C or immersed in RNA*later*^®^ (Thermo Scientific, Grand Island, NY, USA) overnight before being stored at −80 °C until analysis. The study protocol was approved by the Institutional Animal Care and Use Committee (IACUC) at Brooklyn College.

### Analytical measurements

For analytical measurement of placental samples, we excluded dams with a litter size lower than 5 or higher than 10 to prevent the potential confounding effect of litter size on metabolic parameters. Two samples were randomly selected from each litter unless specified otherwise. Researchers were blinded to study grouping when conducting experiments.

### Embryo sexing

The sex of all fetuses was determined by PCR of a sequence specific to the mouse sex-determining region Y (*Sry*) gene on the Y chromosome according to a published method^29^.

### Non-esterified fatty acid (NEFA) measurements

Maternal NEFAs were measured with the HR Series NEFA-HR(2) colorimetric reagents (Wako Diagnostics, Richmond, VA, USA) according to the manufacturer’s instructions.

### Insulin measurement

Maternal serum insulin levels were measured with enzyme-linked immunosorbent assay (ELISA) kits (ALPCO, Salem, NH, USA) according to the manufacturer’s instructions.

### Triglyceride measurements

Placental and maternal serum triglyceride concentrations were quantified with the Triglyceride Colorimetric Assay Kit (Cayman, Ann Arbor, MI, USA) according to the manufacturer’s instructions.

### Glycogen measurements

Fetal liver glycogen was quantified with the Glycogen Fluorometric Assay Kit (Cayman) according to the manufacturer’s instructions.

### Choline measurements

A weighed portion of liquid nitrogen-pulverized liver samples (about 50 mg) was used for choline quantification. Measurements of choline and its derivatives were conducted using the LC–MS/MS methodology^30^. One fetal liver per dam and six dams from each group were randomly chosen for choline quantification.

### RNA extraction and quantitative real-time PCR

RNA extraction and quantitative real-time PCR were conducted using published SYBR-green-based real-time PCR methods^15^. Data were expressed as the fold difference of the gene of interest relative to the housekeeping gene, beta-actin (*Actb*) and compared using the ΔΔCt method^31^. All primers used were published previously^15, 19^. Expression of the following genes was analyzed: genes involved in choline metabolism including betaine-homocysteine *S*-methyltransferase 1 (*Bhmt1*), *Chdh*, choline-phosphate cytidylyltransferase A (*Pcyt1a*), and phosphatidylethanolamine *N*-methyltransferase (*Pemt*); genes involved in placental growth including insulin-like growth factor I receptor (*Igf1r*) and *Igf2*; genes affecting fat and glucose transport including fatty acid transporter 1 (*Fatp1*) and glucose transporter 1 (*Glut1*); and genes participating in de novo lipogenesis including acetyl-CoA carboxylase (*Acc*) 1 and 2, carbohydrate-responsive element-binding protein (*Chrebp1*), fatty acid elongase 5 (*Elovl5*), fatty acid desaturase 1 (*Fads1*), fatty acid synthase (*Fasn*), stearoyl-CoA desaturase-1 (*Scd1*), and sterol regulatory element-binding protein 1, isoform C (*Srebp1c*), as well as diacylglycerol O-acyltransferase 1 (*Dgat1*) which mediates triglyceride synthesis and microsomal triglyceride transfer protein (*Mttp*) that is involved in lipoprotein assembly.

### Statistical analyses

General linear models (GLMs) were constructed to assess the differences in the dependent variables (e.g., embryonic weight, placenta weight, and gene expression) with HF and BS as well as their two-way interaction as independent variables. Post hoc pairwise comparisons were conducted if the interaction term has a *P* value ≤ 0.1. Tukey’s HSD tests were used to correct for multiple comparisons. For the assays in which multiple embryos or placentas from the same dam were analyzed, the dam was adjusted in the model as a random factor. Fetal sex and litter size were also included in the model as independent variables if they significantly modified the dependent variable. Dependent variables deviating from the normal distribution were logarithmically transformed before analysis. A *P* value < 0.05 was considered as significant. A *P* value < 0.1 was considered as a trend to be significant. *P*_D_, *P*_S_, and *P*_I_ represent the *P* values of HF feeding, BS, and their interaction, respectively. Values are presented as means ± standard error of mean (SEM).

## Results

### Food and betaine intake

Maternal HF feeding significantly decreased food intake yet increased total calorie consumption due to the higher calorie density in the HF diet, as previously reported^15^. Maternal BS did not modify food intake or calorie consumption (data not shown). HF and BS interacted to affect the volume of water consumed each week (*P*_I_ = 0.03). Pairwise analysis suggests that the NF-BS (22.8 ± 0.8 mL) group had higher water consumption than the HF-BS group (20.2 ± 0.5 mL, *P* = 0.03), while water consumption in the NF-BC (20.2 ± 0.4 mL) and HF-BC (21.2 ± 0.5 mL) groups was not significantly different. With this difference in water intake, the NF-BS group received 1.94 ± 0.06 mM betaine/week, 13% higher than the 1.72 ± 0.05 mM betaine/week received by the HF-BS group.

### Maternal weight, glucose tolerance, and biomarkers

**Fig. 2:**
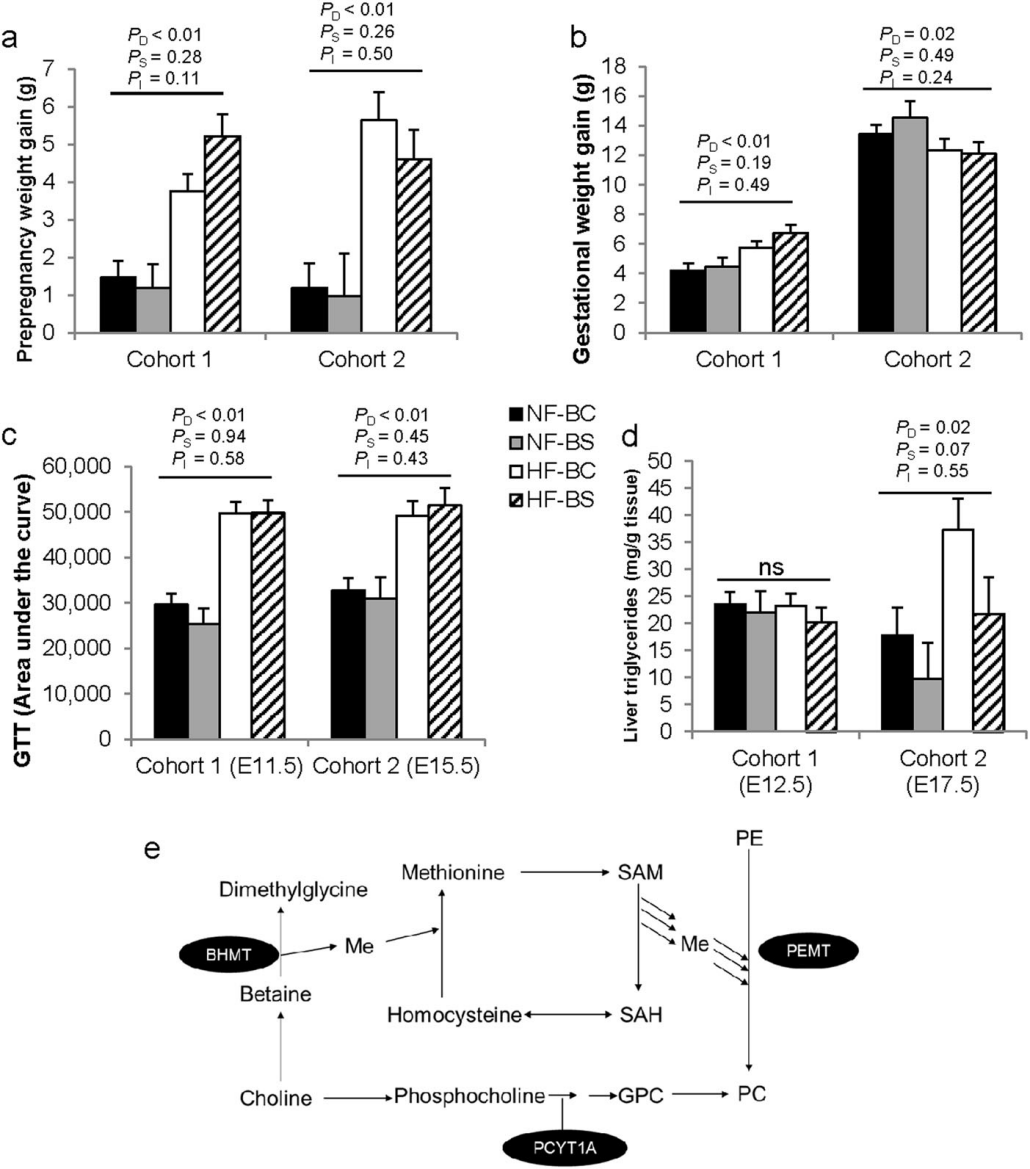
Weight gain and intraperitoneal glucose tolerance (IGT) of dams fed different diets. **a** Prepregnancy weight gain before timed-mating. **b** Weight gain during gestation. **c** The area under the curve (AUC) of the IGT tests at E11.5 or E15.5. **d** Maternal liver triglyceride concentrations. **e** Pathways and enzymes of choline metabolism. Sample sizes of the groups are specified in Fig. 1. NF-BC (solid bars), NF-BS (shaded bars), HF-BC (open bars), and HF-BS (hatched bars). Data were analyzed using the general linear model. *P*_D_, *P*_S_, and *P*_I_ represent the *P* values of HF feeding, betaine supplementation, and their interaction, respectively. Post hoc pairwise comparisons were conducted with Tukey’s HSD correction if *P*_I_  ≤ 0.1. Values are mean ± standard error of mean (SEM); different letters (**a** versus **b**) indicate *P* < 0.05 in the pairwise analysis. ns not significant, BC no betaine control, BS betaine supplemented, HF high-fat diet, NF normal-fat diet

We previously reported greater pre-pregnancy weight gain of the HF-fed dams compared to the NF dams^19^ after either 4 or 6 weeks of HF feeding (*P*_D_ < 0.01). This weight difference was not modified by BS (Fig. 2a). Weight gain during pregnancy was higher at E12.5 (*P*_D_ < 0.01) but lower at E17.5 (*P*_D_ = 0.02) in the HF versus NF groups (Fig. 2b). BS did not modify gestational weight gain at either time point. Glucose tolerance at both E11.5 and E15.5 was worsened by maternal HF feeding (*P*_D_ < 0.01), yet was not modified by BS (Fig. 2c).

We have previously found that maternal HF feeding did not affect liver weight, serum triglyceride, FFA, or serum insulin levels at either time point, but did increase abdominal fat weight (*P*_D_ < 0.01) at both time points^15, 19^. BS did not modify these biomarkers (data not shown). Maternal liver triglyceride content was not affected by HF feeding or BS at mid-gestation (E12.5), but was increased by HF feeding at E17.5 (*P*_D_ = 0.02) and tended to be mitigated by BS (*P*_S_ = 0.07) (Fig. 2d).

We measured betaine content in the liver of the dams at E12.5. BS led to significantly higher hepatic betaine content compared to untreated control (*P*_S_ *<* 0.01) (Table 1). Although the oxidation of choline to betaine is irreversible, betaine may still contribute to the liver choline pool by providing methyl groups for the de novo synthesis of choline via the PEMT pathway, in which the betaine-derived methyl groups are used to sequentially methylate phosphatidylethanolamine-forming phosphatidylcholine (PC) that releases choline after phospholipase digestion (Fig. 2e). Therefore, we also measured the concentrations of choline and its other derivatives. BS and HF interacted to affect free choline concentrations (*P*_I_ = 0.01). Pairwise comparison suggests that the HF-BS group had significantly higher free choline levels than the other three groups (*P* < 0.05). BS also increased methionine concentrations compared to BC (*P*_S_ = 0.04). Phosphocholine (an intermediate metabolite in the Kennedy pathway of PC synthesis) content was significantly decreased (*P*_D_ = 0.04) and PC content tended to be decreased (*P*_D_ = 0.09) by HF feeding, suggesting that HF feeding may have reduced PC synthesis in the maternal liver. BS did not modify the content of phosphocholine or PC.

**Table 1:** Maternal liver betaine and choline derivatives^a^

nmol/g tissue	NF-BC	NF-BS	HF-BC	HF-BS	*P*_D_	*P*_S_	*P*_I_
Betaine	291 ± 114	727 ± 140	231 ± 114	826 ± 140	0.88	<0.01	0.54
Choline	171 ± 23^b^	131 ± 28^b^	164 ± 23^b^	270 ± 28^c^	0.02	0.21	0.01
Methionine	97 ± 13	134 ± 16	99 ± 13	129 ± 16	0.91	0.04	0.81
Glycerophosphocholine	328 ± 27	271 ± 33	305 ± 27	289 ± 33	0.94	0.24	0.51
Phosphocholine	280 ± 89	441 ± 109	105 ± 89	176 ± 109	0.04	0.24	0.66
Phosphatidylcholine	15,209 ± 1603	15,370 ± 1964	17,997 ± 1603	19,142 ± 1964	0.09	0.72	0.79

^a^Different diets were fed to dams from 4 to 6 weeks before timed-mating to gestational day 12.5 or 17.5. Data were analyzed using the general linear model. Post hoc pairwise comparisons were conducted if *P*_I_ ≤ 0.1. The Tukey’s HSD test was used to correct for multiple comparisons. Different letters (b versus c) indicate *P* < 0.05 in the post hoc analysis. Values represent means ± standard error of mean (SEM). BC no betaine control, BS betaine supplemented, HF high-fat diet, NF normal-fat diet, *P*_D_ the *P* value of HF feeding, *P*_S_ the *P* value of BS, *P*_I_ the *P* value of interaction

### Placental and embryonic outcomes

**Fig. 3:**
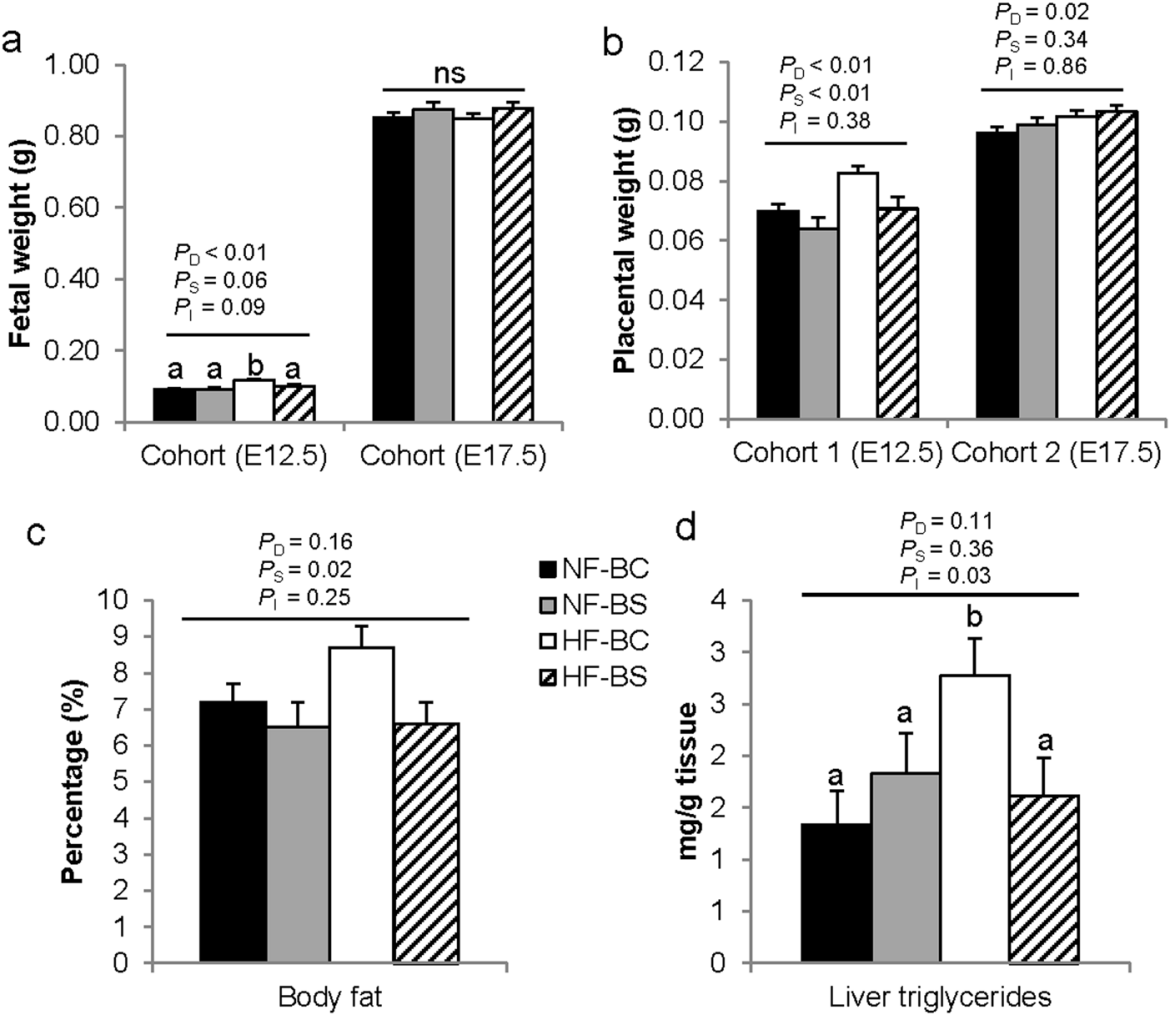
Fetal and placental growth markers of dams fed different diets. **a** Fetal weight at E12.5 or E17.5. **b** Placental weight at E12.5 or E17.5. **c** Percent of body fat at E17.5. **d** Fetal liver triglyceride concentrations at E17.5. *n* = 2 per dam and 6 dams per group; NF-BC (solid bars), NF-BS (shaded bars), HF-BC (open bars), and HF-BS (hatched bars). Data were analyzed using the general linear model. *P*_D_, *P*_S_, and *P*_I_ represent the *P* values of HF feeding, betaine supplementation, and their interaction, respectively. Post hoc pairwise comparisons were conducted with Tukey’s HSD correction if *P*_I_ ≤ 0.1. Values are mean ± standard error of mean (SEM); different letters (**a** versus **b**) indicate *P* < 0.05 in the pairwise analysis. ns not significant, BC no betaine control, BS betaine supplemented, HF high-fat diet, NF normal-fat diet

Our previous report has shown that maternal HF feeding increased placental and fetal weight which was normalized by maternal CS at E12.5^15^. Similar effects were observed with BS. HF tended to interact with BS to influence fetal weight at E12.5 (*P*_I_ = 0.09). Pairwise comparisons demonstrate that fetal weight was higher in the HF-BC group compared to the other three groups (*P* < 0.01) (Fig. 3a). HF increased (*P*_D_ < 0.01) while BS decreased (*P*_S_ *<* 0.01) placental weight at E12.5 (Fig. 3b). At 17.5, neither HF nor BS affected fetal weight (Fig. 3a). However, HF feeding continued to increase (*P*_D_ < 0.01) placental weight which was not modified by BS (Fig. 3b).

We further examined body fat content of fetuses. However, we were not able to assess fetal body fat at E12.5 because the content was below the detection limit of the assay kit. At E17.5, despite the lack of difference in fetal weight, maternal BS significantly lowered the percent body fat of the fetuses (*P*_S_ = 0.02) (Fig. 3c). Maternal HF feeding and BS interacted to affect hepatic triglyceride content (*P*_I_ = 0.03). HF feeding increased hepatic triglyceride content of the fetuses in the HF-BC (*P* < 0.03) but not the HF-BS (*P* = 0.94) group versus NF-BC (Fig. 3d). Liver glycogen concentrations of fetuses were not significantly different among the groups (*P* > 0.05) (data not shown).

**Fig. 4:**
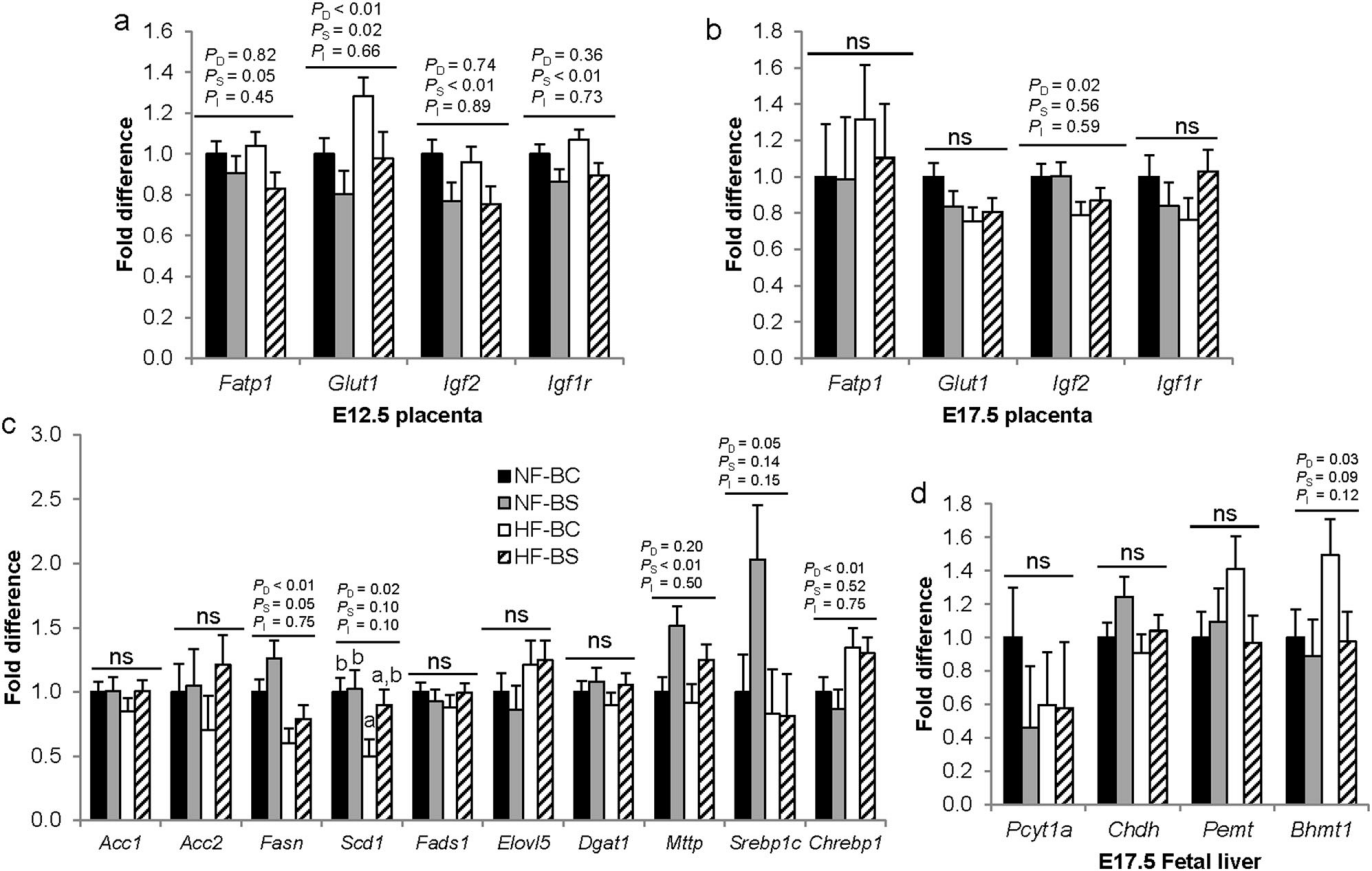
Placental and fetal liver mRNA expression. Placental transporter mRNA abundance at E12.5 (**a**) and E17.5 (**b**). **c** Fetal liver mRNA abundance of genes involved in lipid metabolism at E17.5. **d** Fetal liver mRNA abundance of genes involved in choline and betaine metabolism. *n* = 2 per dam and 6 dams per group; NF-BC (solid bars), NF-BS (shaded bars), HF-BC (open bars), HF-BS (hatched bars). Data were analyzed using the general linear model. *P*_D_, *P*_S_, and *P*_I_ represent the P values of HF feeding, betaine supplementation, and their interaction respectively. Post hoc pair-wise comparisons were conducted with Tukey’s HSD correction if *P*_I_  ≤ 0.1. Values are mean ± standard error of mean (SEM); different letters (**a**, **b**) indicate *P* < 0.05 in the pairwise analysis. ns not significant. *Acc* acetyl-CoA carboxylase, *Acox1* peroxisomal acyl-coenzyme A oxidase 1, *Bhmt* betaine–homocysteine *S*-methyltransferase, *Chdh* choline dehydrogenase, *Chrebp1* Carbohydrate-responsive element-binding protein, *Dgat1* diacylglycerol *O*-acyltransferase 1, *Elovl5* fatty acid elongase 5, *Fasn* fatty acid synthase, GPC glycerophosphocholine, Me methyl group, *Mttp* microsomal triglyceride transfer protein, Pcyt1a choline-phosphate cytidylyltransferase A, PC phosphatidylcholine, PE phosphatidylethanolamine, *Pemt* phosphatidylethanolamine *N*-methyltransferase, *Scd1* stearoyl-CoA desaturase-1, *Srebp1* sterol regulatory element-binding protein 1, BC no betaine control, BS betaine supplemented, HF high-fat diet, NF normal-fat diet

Since fetal weight and body fat content can be influenced by maternal macronutrient transport through the placenta and de novo lipogenesis of the fetus itself, we examined metabolic gene expression in the placenta and fetal liver. We previously reported that maternal CS decreased transporters *Glut1* and *Fatp1* mRNA expression during HF feeding at E12.5^15^. In this study, we identified similar effects that maternal BS led to lower*Fatp1* (*P*_S_ = 0.05) expression than BC. HF feeding increased (*P*_D_ < 0.01) while BS reduced (*P*_S_ = 0.02) *Glut1* expression (Fig. 4a). Maternal BS also lowered (*P*_S_ < 0.01) both placental *Igf2* and *Igf1r* expression in the BS versus BC groups at E12.5. However, the expression levels of these transporters and growth factors were not affected by HF or BS at E17.5, except for *Igf2*. HF feeding led to lower *Igf2* expression (*P*_D_ = 0.02) (Fig. 4b).

We also examined fetal hepatic mRNA expression among the groups at E17.5. Unlike maternal CS, BS did not downregulate expression of genes involved in de novo lipogenesis including *Acc2*, *Fads1*, *Elovl5* or their upstream regulators *Srebp1c* and *Chrebp1* (*P*_S_ > 0.05) versus BC (Fig. 4c). Moreover, HF feeding decreased (*P*_D_ < 0.01) while BS increased (*P*_S_ = 0.05) the expression of *Fasn*, which promotes fatty acid synthesis. HF-BS also obviated the reduction in *Scd1*, another fatty acid synthesizing gene, in the HF-BC group versus the NF-BC group (*P*_I_ = 0.1). HF or BS did not affect the expression of *Dgat1* which mediates fatty acid esterification either. However, maternal BS significantly upregulated (*P*_S_ < 0.01) mRNA expression of *Mttp* compared to BC, which may facilitate lipoprotein assembly and prevent triglyceride accumulation in the fetal liver.

We explored the changes in betaine and choline metabolism in the fetal liver that may have mediated the changes in lipid metabolism at E17.5. HF increased (*P*_D_ = 0.03), whereas BS tended to decrease (*P*_S_ = 0.09) *Bhmt1*expression (Fig. 4d). Moreover, both HF feeding (*P*_D_ = 0.04) and BS (*P*_S_ < 0.01) increased betaine content in fetal livers. Therefore, the HF-BS group had the highest betaine concentrations as compared to the other three groups (NF-BC: 750 ± 263, NF-BS: 1354 ± 354, HF-BC: 970 ± 299, HF-BS: 2699 ± 299 nmol/g tissue). Other metabolites or genes involved in choline metabolism were not affected by HF or BS.

## Discussion

In this study, we have found that maternal BS normalizes the weight gain of fetuses from dams fed a HF diet at mid-gestation as well as decreases percent body fat and prevents hepatic triglyceride over accumulation at late gestation. These phenotypes were consistent with observations in our previous studies on maternal CS. However, gene expression and metabolite analyses indicate that the metabolism of the maternal and fetal dyad may be regulated differently by choline and betaine at different gestational time points.

### Maternal BS was as effective as CS on growth and adiposity outcomes of fetuses from HF dams

This study is a continuation of our earlier studies where we demonstrated that supplementation of choline prevents fetal overgrowth and excess adiposity in dams demonstrating phenotypes that resemble maternal obesity and GDM^15, 19^. We also observed that betaine was the only choline derivative with increased concentrations in maternal livers after CS^15^. Betaine is formed from choline via an irreversible oxidation reaction and it is an intermediate in the pathway where choline participates as a methyl donor. In the current study, we found that maternal BS was as effective as CS in reducing fetal or placental weight gain during mid-gestation (E12.5) in the HF dams. This finding provides further evidence that the influence of CS on fetal growth at this time point may be achieved via its increased oxidation to betaine and the metabolic processes associated with betaine.

Nevertheless, the exact mechanism by which betaine influences macronutrient metabolism and growth outcomes of the fetus remain elusive. Maternal CS lowers the expression of glucose and fat transporters as well as their upstream regulator and placental growth promoter *Igf2* in the placenta^15^. The current study also demonstrates that maternal BS reduced the expression of both *Glut1* and *Fatp1*. Moreover, maternal BS had a strong effect on reducing the expression of both *Igf2* and its receptor *Igf1r*, regardless of HF or NF feeding. Such influence on *Igf1r* expression was unique for BS and not observed with maternal CS. IGF2 is a major growth factor that dictates both placental and fetal sizes during gestation^32^. IGF1R mediates the action of IGF2. The IGF signaling system positively regulates macronutrient transport in the placenta^33, 34^. Collectively, current data indicates a plausible mechanism that choline or BS prevents fetal overgrowth via the downregulation of genes involved in placental nutrient transport and placental growth factors.

*Igf2* is an imprinted gene with its expression tightly regulated by DNA methylation levels of differentially methylated regions^35^. *Igf1r* expression is also reported to be influenced by DNA methylation^36^. Since a major role of betaine is to serve as a methyl donor, increasing its availability during gestation may have increased methyl group provision for DNA methylation of *Igf2* and *Igf1r*, or other placental growth factors, thereby decreasing their expression and macronutrient transport through the placenta that they regulate.

Since betaine is formed via irreversible oxidation of choline, it is not a direct source of choline for PC synthesis. However, betaine may still contribute to the PC pool by providing methyl groups for the sequential methylation of phosphatidylethanolamine to form PC via the PEMT pathway^37^. Therefore, the possibility that BS alters PC signaling^38, 39^ which influences other nutrient sensing mechanisms such as the mechanistic target of rapamycin (mTOR) to affect placental transport^16^ cannot be ruled out.

### Maternal BS prevented excess fetal adiposity potentially via a different mechanism than CS

Both BS and CS prevented the increase in fetal fat percentage and TG over accumulation at E17.5. Interestingly, betaine attained such effects without altering the expression of lipogenic genes *Acc2*, *Fads1*, *Elovl5* or their upstream regulators *Srebp1c* and *Chrebp*, which were all downregulated by CS^19^. BS even mitigated the decrease in *Fasn* and *Scd1* expression observed in the HF-BC group versus NF-BC, which further attenuated the adjustment to decrease fatty acid synthesis in response to HF feeding. These results indicate that mechanisms other than a reduction in de novo lipogenesis mediate the influence of maternal BS on fetal adiposity. Fetal hepatic *Mttp* expression was higher in the BS groups than the BC groups, while its expression was not altered by maternal CS^19^. The upregulation of *Mttp* might explain the reduced triglyceride content in the HF-BS fetal livers since it is required for the assembly and secretion of VLDLs from the liver. However, the role of MTTP in whole body adiposity is uncertain, since *Mttp* deficiency in mice does not affect total adiposity or glucose tolerance^40^.

The more than twofold increase in fetal liver betaine content in the HF-BS group provides a vast pool of methyl group for methylation reactions. The lower *Bhmt1* expression by BS is also consistent with the enhanced use of betaine for homocysteine remethylation to methionine since SAM, the universal methyl donor derived from methionine, has an inhibitory effect on BHMT1^41^. Notably, one carbon metabolism consumes energy thereby reducing anabolism^42^. Moreover, methylation of DNA and proteins alters expression and activity of metabolic enzymes, thereby influencing lipid metabolism in the fetus. Maternal BS has been shown to alter DNA methylation of genes involved in cholesterol metabolism such as LDL receptor, gluconeogenesis such as *Pck*, as well as DNA methylation and expression of glucocorticoid receptor in the brain and its miRNA regulators, which can influence whole body energy homeostasis in stress^12, 13, 43^. The role of betaine in activating AMP-activated kinase (AMPK) provides another possible mechanism that might contribute to the prevention of triglyceride accumulation. AMPK regulates ACC and fatty acid synthase, rate limiting enzymes in fatty acid synthesis in the liver^9^. Others also demonstrate that mitochondrial betaine degradation is important for increasing energy expenditure which enhances lipid catabolism and prevents hepatic accumulation^3^. In summary, although maternal BS did not lower lipogenic gene expression or placental transport at late gestation, the increase in betaine in the liver may be sufficient to reduce liver steatosis and whole body fat metabolism via other mechanisms.

It should be noted that the attenuating effect of BS on fetal growth and hepatic fat accumulation was only observed in the HF but not NF groups, suggesting an interaction between HF and BS. This interaction may be related to the increased utilization of betaine as a methyl donor or a lipotrope during metabolic disturbance^44–50^. Therefore, BS may aid in meeting the higher demand for betaine in the HF fed dams.

### The temporal differences in placental and fetal response to HF and BS

In our study another interesting phenomenon that emerged was the temporal-specific effect of betaine on fetal growth and placental transport. The reduction of fetal growth and nutrient transport by BS at E12.5 did not sustain into late gestation, suggesting that placental and fetal response to the maternal environment depends on the specific stage of gestation. Previous studies in mice reported similar temporal-specific response in fetal growth. Calorie restriction by 50% during E1.5–11.5 led to placental weight reduction in early gestation, yet this weight reduction was reversed at E18.5^51, 52^. Over-nutrition induced by a high-sugar, HF diet reduced feto-placental growth at E16, yet fetal weight was normalized at E19^53^.

The development of placental networks regulating nutrient transfer is maximized by mid-gestation. Placental transport appears to be especially sensitive to maternal nutrition during this stage of rapid proliferative development of the organ^54^. Placental fatty acid transporter expression is associated with maternal cholesterol and triglyceride status at mid-gestation in ewes^55^. Under-nutrition of mouse dams reduced GLUT1 expression at E16 but not at E19^56^. Supplementing choline to dams decreased the expression of GLUT1 at E15.5 but not at E18.5^57^. Late gestation is characterized by the growth spurt of the fetus related to lipid accretion, while placental growth is lessened^56^. The placenta adapts itself to the need of the fetus. The fetal liver also plays a more important role in regulating its own metabolism at this stage^13^. Our observation that placental nutrient transporters were only altered at mid-gestation by maternal HF or BS corroborates the aforementioned studies.

### BS did not modify maternal endpoints of the HF dams

There were differences in the effect of BS and CS on the metabolic outcomes of HF dams. Although both BS and CS had limited effects on maternal metabolism versus placental or fetal outcomes at either time points, CS seemed to improve glucose tolerance while paradoxically promoted hyperinsulinemia and increased circulating FFAs regardless of HF feeding at E17.5^19^. BS did not affect any of the above endpoints, although its effect on reducing hepatic triglyceride accumulation of the dams was more pronounced than CS. BS also seems to be more effective in altering concentrations of one carbon metabolites such as methionine and free choline in maternal livers. These observations again suggest that BS and CS, while having shared pathways of influence, also have differential impacts on metabolism via the different biochemical processes that they participate in.

## Conclusions

This study demonstrates that maternal BS produced similar phenotypes to CS on placental and fetal growth and adiposity in response to maternal obesity in mice. However, the underlying mechanisms of action may be different. Their impacts on fetal outcomes are also temporal and tissue specific. This study provides proof of concept evidence that betaine may be further explored as adjuvant therapy to current nutrition intervention methods for the improvement of fetal outcomes during maternal obesity and GDM.
